# Effect of Ethanol Extract of Propolis on Paper Packaging Material

**DOI:** 10.3390/ma19184016

**Published:** 2026-09-21

**Authors:** Stanka Damyanova, Darina Georgieva, Iliana Kostova, Iliyana Nikolova, Vanya Prodanova-Stefanova, Ivelin Dimitrov, Albena Stoyanova, Dimitrina Todorova, Nikolay Yavorov

**Affiliations:** 1 Department of Biotechnologies and Food Technologies, University of Ruse “Angel Kanchev”, Razgrad Branch, 7200 Razgrad, Bulgaria; dsgeorgieva@uni-ruse.bg (D.G.); ikostova@uni-ruse.bg (I.K.); inikolova@uni-ruse.bg (I.N.); 2 Technical Faculty, South-West University “Neofit Rilski”, 2700 Blagoevgrad, Bulgaria; v_t_p@abv.bg; 3 Sliven Faculty and College, Technical University of Sofia, 8800 Sliven, Bulgaria; 4 Department of Engineering Technology and Metal Cutting Machines, University of Ruse “Angel Kanchev”, 7017 Ruse, Bulgaria; ivelin8652@gmail.com; 5 Department of Tobacco, Sugar, Vegetable and Essential Oils, University of Food Technologies, 4002 Plovdiv, Bulgaria; aastst@abv.bg; 6 Department of Pulp, Paper and Printing Arts, Faculty of Chemical Technology, University of Chemical Technology and Metallurgy, 1797 Sofia, Bulgaria; todorova.dimitrina@uctm.edu (D.T.); nyavorof@uctm.edu (N.Y.)

**Keywords:** ethanol extract of propolis, treating paper, paper-based packaging materials, antimicrobial activity

## Abstract

Propolis is a natural, accessible, and valuable bee product belonging to the group of so-called bioproducts. Its composition varies significantly depending on a number of factors, resulting in a wide range of data. The aim of this work is to investigate the antimicrobial effect of propolis extract, prepared with 70% ethanol, on packaging paper. Three propolis samples from Bulgaria were used: from the Northeastern region—harvest 1993 and 2023—and from the Central region—harvest 2023. Paper samples from a cellulose blend only and a base paper with improved hydrophobicity from cellulose blends of two samples of bleached kraft woodfree pulp are used for the analysis. The antimicrobial activity of ethanol extract from propolis is tested against Gram-positive bacteria *Staphylococcus aureus*, *Staphylococcus epidermidis*, and *Bacillus cereus*, Gram-negative bacteria *Pseudomonas aeruginosa*, a yeast, *Candida albicans,* and a mold, *Fusarium verticillioides*. The growth of microbial colonies is observed for up to 120 h. The results show over 90% effect when treating paper with ethanol extract of propolis, which is an indication that it has significant potential for inclusion in antimicrobial active packaging materials.

## 1. Introduction

Ensuring food quality and safety for the protection of human health is an important task for the food industry. In this context, market requirements for food packaging are becoming increasingly stringent. Packaging protects the product from external contamination and is responsible for its preservation; it also serves as a marketing tool for communication and convenience [1]. In general, packaging is classified as passive or functional (smart), each with specific mechanisms of action and functional objectives. Conventional passive packaging acts as a physical barrier against oxygen, moisture, light, and mechanical impacts. Such packaging does not respond to changes in the food environment and does not inhibit microbiological or oxidative processes [2].

Modified Atmosphere Packaging (MAP) is a method for extending shelf life by regulating the composition of the gaseous environment within the package. The most commonly applied techniques include vacuum packaging, nitrogen flushing, carbon dioxide injection, and others. Reduced oxygen content acts as a barrier to aerobic microorganisms and oxidative processes. Elevated carbon dioxide concentrations exert a bacteriostatic effect; however, anaerobic spoilage processes may occur, posing a risk of toxicological contamination. Therefore, these methods are often combined with other preservation approaches (e.g., temperature control) [3].

The latest trends in packaging involve functional active packaging, which interacts deliberately with the food product or the internal atmosphere of the package. Such packaging may release active compounds or adsorb undesirable components, thereby extending shelf life and improving food safety [4]. Active packaging includes scavenger and releasing systems and may possess both antimicrobial and antioxidant properties. “Scavenger” systems selectively remove moisture, oxygen, or ethylene. Moisture reduction limits the growth of microbial organisms, oxygen removal suppresses the growth of aerobic microbial species, and absorption of ethylene released by fruits and vegetables delays their ripening and softening. These systems are used for products sensitive to oxidation and function through chemical reactions, adsorption, or enzymatic mechanisms [1].

In functional packaging based on the controlled release of substances from the packaging material, antimicrobial agents are most commonly used to prevent microbial spoilage of the food product. Antimicrobial packaging consists of a polymer matrix into which an antimicrobial agent is incorporated, inhibiting the growth of microorganisms through diffusion or direct contact. The mechanisms of action include the disruption of microbial cellular structures [5]. Recently, there has been increasing interest in the use of natural bioactive substances and sustainable development models in the packaging industry [6,7].

The following agents exhibit antimicrobial potential in active packaging: metal ions, such as silver, copper, gold, and platinum; metal oxides (TiO_2_, ZnO, MgO); essential oils, including thyme, oregano, clove, lemon balm, and others; plant extracts, including grape seed, green tea, pomegranate peel, rosemary, garlic, ginger, and others; polysaccharides (chitosan); essential oil isolates (thymol and carvacrol); peptides, including nisin and lactoferrin; enzymes, including peroxidase and lysozyme; and synthetic antimicrobial agents, including EDTA, propionic acid, benzoic acid, and sorbic acid [8,9].

Growing attention has been directed toward the utilization of natural compounds in food product innovation and functional packaging design. Plant extracts and essential oils are especially noteworthy due to their biologically active components conferring antimicrobial and antioxidant properties. Extracts from grapefruit, grape, and pomegranate seeds, cinnamon bark, horseradish root, and clove buds have been incorporated into packaging materials, providing microbial inhibitory activity and protection against undesirable microflora [10]. Similarly, essential oils from herbs integrated into biodegradable packaging for broccoli effectively inhibit *Salmonella typhimurium*, *Escherichia coli*, and *Listeria monocytogenes* [11].

Controlled-release systems are of particular importance in the development of active packaging, as the active substance migrates from the polymer matrix to the surface of the food product. The factors influencing this process include the diffusion properties of the matrix, the degree of swelling, interactions between the polymer carrier and the active substance, as well as environmental factors such as temperature and humidity [3].

In recent years, there has been growing interest in biodegradable and bioactive packaging based on biopolymers, such as chitosan, starch, gelatin, polylactic acid, and cellulose derivatives. The combination of such matrices with natural antimicrobial and antioxidant agents is considered a sustainable strategy for the development of functional packaging materials [3].

Paper, as a biodegradable material, can serve as a carrier for bioactive compounds that enhance its barrier properties [12,13,14]. Three types of packaging paper were studied—bleached, unbleached and recycled with a coating of three essential oils: from dill (*Anethum graveolens* L.) [15], from coriander (*Coriandrum sativum* L.) [16], and from clary sage (*Salvia sclarea* L.) [17]. Treatment with these essential oils demonstrated excellent protection against bacteria, molds, and yeasts.

Although propolis is not of plant origin, it is a natural product with a rich chemical composition and biological activity, for example, antimicrobial [18,19,20,21,22,23,24,25,26,27,28,29], antioxidant [29] and others [18,30,31,32], which justify its wide applicability. The studies demonstrated that propolis exhibits antimicrobial activity against a broad range of pathogenic and opportunistic microorganisms, including Gram-positive and Gram-negative bacteria, yeasts, and fungi. Many of them can be detected on the surfaces of various food products, fruit and vegetable storage surfaces, processing equipment, and other food contact surfaces. Some of these microbial organisms are associated with foodborne and other infections. Notable examples include the pathogenic Gram-negative bacteria *Escherichia coli*, which may be transferred through contaminated hands and food contact surfaces, and the yeast *Candida albicans*. Propolis is a promising active agent due to its rich content of flavonoids and phenolic acids [33,34,35,36,37,38,39], which exhibit antimicrobial and antioxidant activities [40]. The incorporation of propolis into polymer matrices enables the development of active food packaging with the potential for the controlled release of bioactive components and extension of the shelf life of food products [4]. The characteristic organoleptic properties of propolis, including its distinctive taste and aroma, require careful consideration when integrating it into packaging carriers to ensure compatibility with the organoleptic characteristics of the food [41,42]. This indicates that, for each specific food product intended to be packaged in paper containing propolis, sensory evaluation, including assessment of aroma, odor, and other relevant sensory attributes, should be conducted to ensure that the packaging material does not adversely affect the sensory quality of the food and to support consumer safety.

The aim of the present study was to evaluate the effectiveness of treating paper with extracts of Bulgarian propolis obtained from different geographical regions, with a view to assessing its potential application as a food packaging material and its ability to protect packaged food products against microbial spoilage.

## 2. Materials and Methods

### 2.1. Materials

**Figure 1:**
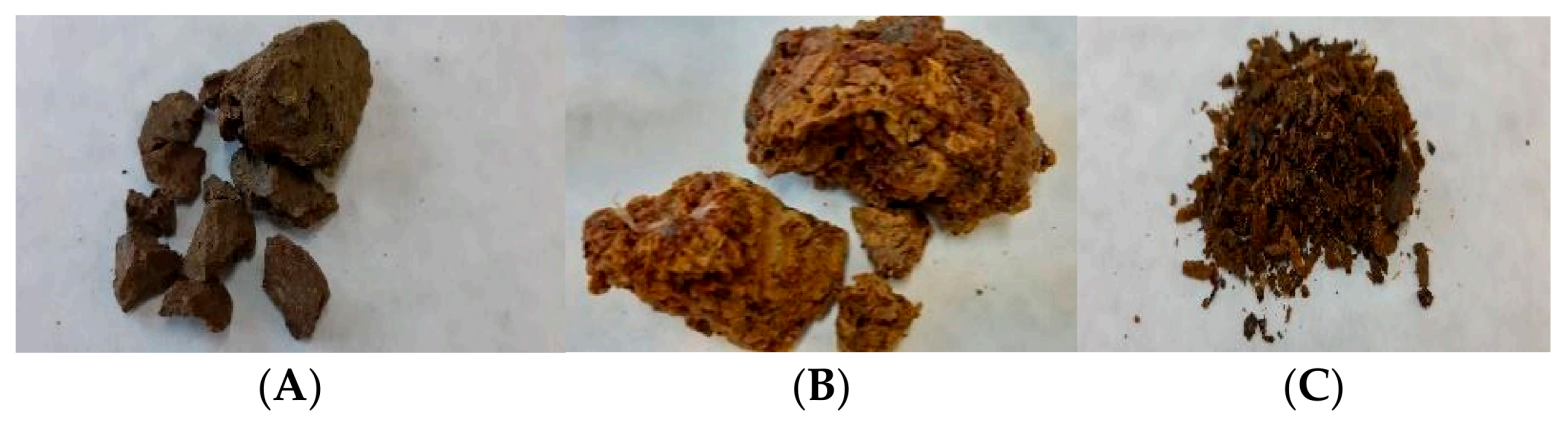
Propolis (authors’ own image): (**A**)—from Northeastern, Bulgaria; (**B**)—from Central Bulgaria; (**C**)—from Northeastern Bulgaria.

Three samples of propolis were used for this study (Figure 1):

− Sample 1: Stored for 30 years, originating from Northeastern Bulgaria (43°32′ N 26°31′ E, altitude 270 m) (Figure 1A);
− Sample 2: Collected in 2023 from Central Bulgaria (42.61°94′418″ N 25.39°29′58″ E, altitude 407 m) (Figure 1A);
− Sample 3: Collected in 2023 from Northeastern Bulgaria (43°32′ N 26°31′ E, altitude 270 m) (Figure 1A).

In the sample stored for 30 years (A), the following groups of compounds were identified: flavanones and flavones (74.85%), followed by hydrocinnamic acids and esters (17.15%), monosaccharides (2.95%), and alcohols (2.47%), with the remaining groups below 2% [43].

Propolis from Central Bulgaria, harvest 2023 (B), contained: flavanones and flavones (55.38%), followed by hydrocinnamic acids and esters (25.69%), monosaccharides (13.59%), benzoic acids (2.51%), alcohols (1.65%), fatty acids (0.88%), and organic acids (0.30%) [44].

Propolis from Northeastern Bulgaria, harvest 2023 (C). The data show that the following ones prevail: flavanones and flavones (35.85%) with the main representatives being squalene (10.51%), 717 dihydrochrysin (5.91%), pinobanksin-3-butenoate (4.92%), galangin (3.77%), and pinobanksin (3.74%). The next group of compounds are hydrocinnamic acids and esters (31.07%) with the main representatives being benzyl-(E)-caffeate (11.27%), caffeic acid isomer 1 (2.71%), caffeic acid isomer 2 (2.67%), and (E)-p-coumaric acid (2.25%). From the monosaccharides (28.72%) group, the main components are glucose (9.27%), manose (7.38%), fructose (6.60%), and galactose (3.49%). Other groups of compounds are fatty acids (1.73%), benzoic acids (1.46%), alcohols (0.90%), and organic acids (0.27%) [45].

The differences in the composition of the three investigated propolis samples may be attributed to their geographical origin, harvest year, and storage duration. The effect of storage is particularly pronounced in Sample 1, which had been stored for more than 30 years in a metal container at room temperature (20 °C), in the dark, inside a wooden cabinet, away from direct sunlight and in the absence of extraneous odors. Sample 3 originated from the same geographical region as Sample 1 but was obtained from a different harvest year. In addition, the foraging conditions of the bees differed between the two sampling periods. Sample 2 originated from a different region of the country, characterized by different plant species, altitude, climatic conditions, and soil properties.

A summary of the major groups of compounds identified in the investigated samples is presented in Table 1. The data demonstrate considerable variation in the relative distribution of the major compound groups, particularly flavanones and flavones, as well as acids and esters, which are considered important contributors to the antimicrobial properties of propolis.

### 2.2. Methods

#### 2.2.1. Preparation of Propolis Extracts

Extraction is an important process aimed at achieving the most complete possible recovery of the components contained in the raw material. The extracts were obtained under the following conditions: solvent 70% ethanol, propolis: solvent ratio 1:7, temperature 20 ± 0.5 °C, for 7 days in the dark. The resulting extracts were filtered and stored in dark containers at room temperature (20 ± 0.5 °C).

The selection of the extraction solvent was based on preliminary unpublished data and was guided by the chemical composition of the investigated propolis samples. A 70% ethanol solution is widely used in pharmaceutical preparations for obtaining tinctures from various plant-derived raw materials. Owing to its polar nature and favorable penetration properties, aqueous ethanol is an effective extraction medium for a broad range of bioactive compounds. The presence of water in 70% ethanol facilitates the penetration of the solvent into the plant-derived matrix, while ethanol promotes the solubilization and extraction of valuable propolis constituents, including flavonoids, phenolic acids, and other polar and moderately polar compounds. Under these extraction conditions, waxes and certain resinous components exhibit limited solubility and are therefore largely retained in the solid matrix rather than being efficiently extracted. It is known that both the dielectric constant of the solvent and its dissipation coefficient have an impact on extraction. Water is known to have a higher dielectric constant (ε at 20 °C = 78.3) than ethanol (ε at 20 °C = 24.5), but its dissipation factor is lower. Therefore, solutions of ethanol with water are used, which affects their physicochemical parameters and selectivity [46].

The extracts were characterized by appearance, color, relative density, and refractive index [43,44,45].

The propolis extract is a mobile liquid with a characteristic odor.

It shows very good antimicrobial effect against the Gram-positive bacteria, Gram-negative bacteria and yeast [43,44,45].

#### 2.2.2. Test Microorganisms

The following microorganisms were used as test cultures: Gram-positive bacteria: *Staphylococcus aureus* ATCC 6538, *Staphylococcus epidermidis* ATCC 12228, and *Bacillus cereus* ATCC 10876 and Gram-negative bacteria: *Pseudomonas aeruginosa* ATCC 9027; yeasts: *Candida albicans* ATCC 10231; and molds: *Fusarium verticillioides*.

The selection of the test microbial strains was based on previous studies, both international and our own, demonstrating that propolis exhibits an antimicrobial effect against a broad range of pathogenic and opportunistic microorganisms, including Gram-positive and Gram-negative bacteria, yeasts, and filamentous fungi. The antimicrobial properties of propolis are directly associated with its rich and diverse chemical composition.

*The colony counting procedure was determined.* Two consecutive ten-fold dilutions of the saline suspensions on the appropriate test culture selective media, with 1 mL of proper dilution of each suspension evenly distributed in four pre-spilled and dried plates, were applied for the spread plate techniques used. The consecutive dilutions’ yield presented 25–250 colonies; calculations were done using Equation (1):

N = ΣC/[(1 × n_1_) + (0.1 × n_2_)]d (1)

where *N*—number of colonies per milliliter or gram, CFU/mL, *ΣC*—sum of all the colonies on all the plates counted, *n*_1_—number of plates in the next lower dilution counted, *n*_2_—number of plates in the highest dilution counted, and *d*—dilution from which the first counts were obtained.

#### 2.2.3. Preparation of Microbial Suspensions

A 24 h culture was prepared from each bacterial test microorganism. The vegetative material was taken with a wire loop and suspended in 10 mL of saline. The suspensions prepared were with a cell concentration of about 10^3^ CFU/mL. Yeast and mold suspensions were analogously prepared, but the yeast cultures used were cultivated for 48 h, while the mold one—for 120 h.

#### 2.2.4. Paper Sample Composition and Preparation

Aiming to investigate propolis extracts’ functionality as an active packaging material, the prepared samples of only the cellulose mixture and base paper with improved hydrophobicity were prepared from the cellulose mixtures of two bleached kraft woodfree pulp samples. The mixture was prepared from softwood pulp (delivered by Svenska Cellulosa Aktiebolaget, Sundsvall, Sweden) and hardwood tree species (delivered by Svilosa AD, Svishtov, Bulgaria) in a ratio of 80:20. The celluloses were refined separately via a laboratory Valley beater method [47], and the Schopper Riegler, °SR (WH-5, Block B, Mayapuri Industrial Area Phase I, Mayapuri, New Delhi, Delhi, India) [48], value of the used cellulose mixture was 30 °SR.

Wet-end chemical additives were added to the cellulose mixture and base paper was obtained by adding chemicals in the following sequence: alkylketendimer (AKD) sizing agent—1% (Kemira^®^ Fennosize KD 157YC, Kemira Oyj, Helsinki, Finland)—and cationic retention additive—0.025% (modified polyacrylamide with molecular weight 11.10^6^ g/mol and charge density +1.05 from Ciba Specialty Chemicals-Ciba^®^ Percol^®^ Co. (Basel, Switzerland).

Laboratory simulation of the paper manufacturing process was carried out with a Rapid-Köthen laboratory sheet-forming apparatus (Birkenau, Germany) [49]. Paper samples with a basic weight of 50 g/m^2^ were obtained and dried at a temperature of 94 °C for 6 min.

#### 2.2.5. Microscopic Analysis

Generally, to study the size and structure of the source fibers and to determine the paper composition, a specific microscopic determination of cellulose fibers and paper materials is used. The morphology and composition of the cellulose fibers were characterized by optical microscopy. A small amount of mechanically pre-milled fibers was placed in a porcelain mortar, treated with 10 mL of 1% NaOH solution, and allowed to stand for 10 min. The fibers were then thoroughly rinsed with distilled water using a fine metal mesh. Three uniformly distributed fiber specimens were prepared on a glass microscope slide from the resulting fiber suspension. A drop of Herzberg’s reagent (Cl–Zn–I) [50] was applied to each specimen, after which the fibers were carefully redistributed to ensure uniform staining. The prepared slides were dried in a laboratory chamber at approximately 50 °C. After cooling to room temperature (20 ± 0.5 °C), each specimen was covered with a coverslip, ensuring the absence of air bubbles and fiber agglomerates. The stained fiber samples were examined using a VisiScope^®^ TL254T1 microscope (VWR, Milano, Italy) equipped with an integrated digital camera at a magnification of 100× (objective: 10×/0.25 E-PLAN; eyepiece: WF10×/20 mm). The acquired micrographs were subsequently processed and analyzed using Optika ProView software (version 2.0).

#### 2.2.6. SEM Analysis

The surface morphology of the developed paper materials was examined using a scanning electron microscope (SEM; EVO 10, Carl Zeiss GmbH, Oberkochen, Germany). SEM observations were performed at an accelerating voltage of 10 kV and a magnification of 500×. Micrographs were acquired for both the untreated paper and the propolis-treated samples to evaluate the morphological changes induced by the surface treatment and to assess the distribution and continuity of the deposited propolis layer.

#### 2.2.7. Paper Sample Characteristics

*Basic Weight, Thickness, Density, Porosity, Smoothness*.

A standard method—ISO 536:2019—was used for the basic weight determination of all the prepared samples [51], followed by thickness determination and density and porosity calculation, as described in the ISO method [52]. The Bekk method [53] was used for smoothness determination.

*Tensile Strength, TEA Index, Elongation and Tear Resistance*.

On a Zwick/Roell tensile testing machine, the tensile strength, TEA Index and elongation at break of the obtained paper samples were measured, according to the ISO standard [54] at a 20 mm/min test speed. Ten probes of each paper specimen with 18 cm length stripes and 1.5 cm in width were used. Tensile strength was determined as the tensile index, Nm/g. The samples were analyzed in the standard atmosphere—23 °C temperature and 50% of relative humidity.

Tear resistance, as described in the ISO standard [55], was performed on an Elmendorf tester and expressed as the tear index (with respect to paper basic weight) in mNm^2^/g. It measures the force required to tear the paper after a cut has already been made. Two parallels were measured for each paper sample.

#### 2.2.8. Treatment of Paper with Propolis Extracts

Paper sheets were cut into 5 × 5 cm squares. Each square was weighed with an accuracy of ±0.0001, immersed in the corresponding propolis extract for 1 min, then drained, dried until the constant weight, and reweighed. The amount of extract absorbed by each paper sample was calculated.

#### 2.2.9. Determination of Antimicrobial Activity

Each treated paper specimen’s square was placed aseptically in a sterile Petri dish. A 0.1 mL aliquot of microbial suspension was applied to each paper sample and spread evenly. The growth of microbial colonies was observed for up to 120 h (5 days). The first sample was tested immediately (24 h), while the others were tested after being kept at room temperature (20 ± 0.5 °C), for 48 and 120 h. Soybean–casein digest agar (Humedia, Kennett Square, PA, USA) was used for bacteria, and Sabouraud dextrose agar (Humedia, Kennett Square, PA, USA) was used for yeasts and molds. Paper without propolis extract applied was used as a control.

The cultures were incubated at 30–35 °C for 24–48 h for bacteria, 48–72 h for yeast, and 120 h for molds.

The effect of the developed paper material on the growth of the test microorganisms was evaluated by comparing the number of grown microorganisms on the treated samples with that of the corresponding control sample [56].

The antimicrobial efficiency of the treated paper specimens was calculated using the Equation (2):

Efficiency (%) = (N_0_ − N_t_)/N_0_ × 100, (2)

where N_0_ is the number of colony-forming units in the control, and N_t_ is the number of colony-forming units in the treated samples.

### 2.3. Statistical Analysis

All experiments were performed in triplicate, with values in the tables and figures averaged, and represented with their mean and standard deviation (SD). The experimental data were subjected to statistical evaluation using analysis of variance (ANOVA) with Statgraphics Centurion XVI Version 16.2.04 software (StatPoint Technologies, Inc., Warrenton, VA, USA), and the significance was defined at *p* < 0.05.

## 3. Results and Discussion

### 3.1. Cellulose Fiber Characterization

**Figure 2:**
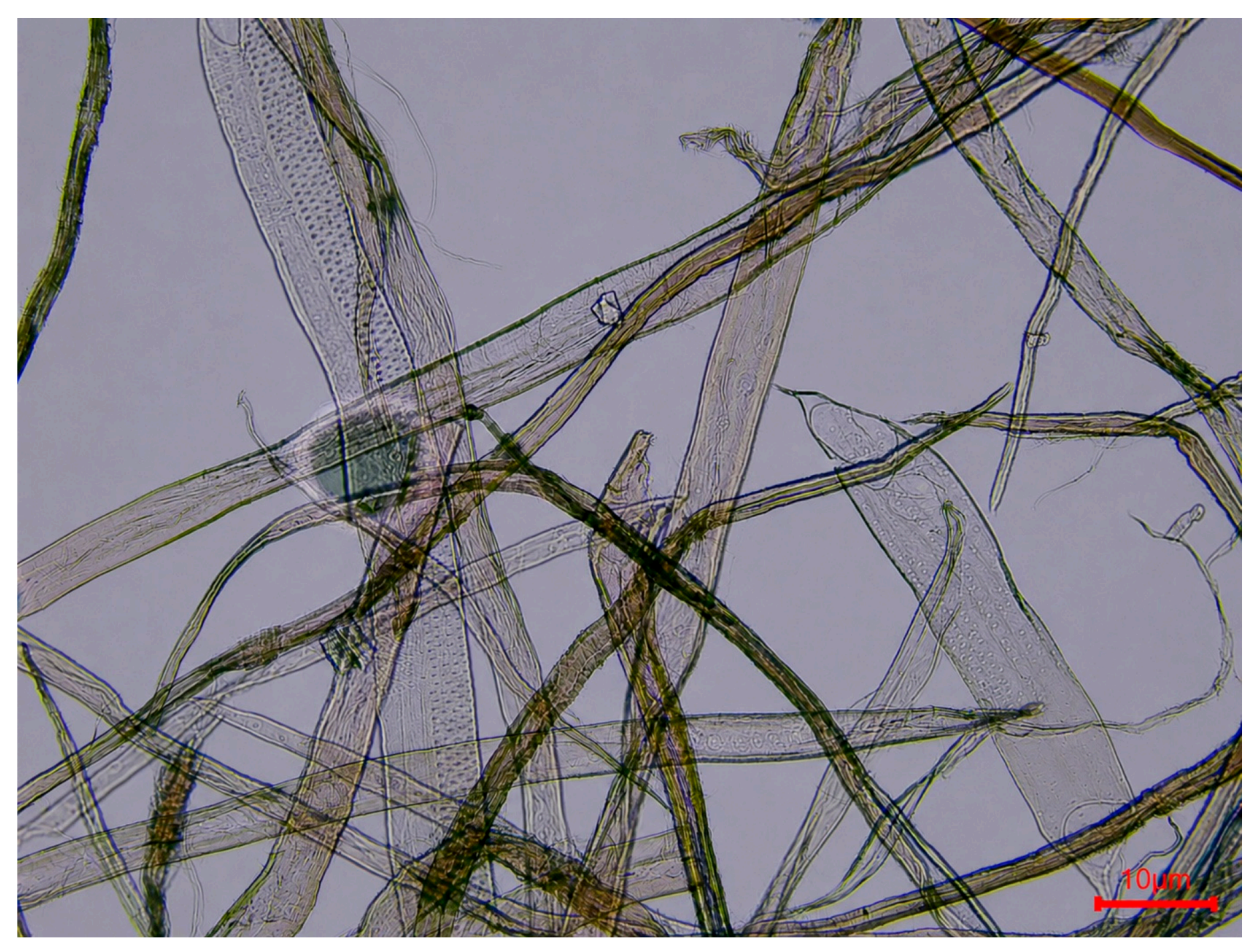
Microscopic photograph of cellulose mixture on the prepared paper samples.

As a wrapping or active propolis-derived extract-developed paper material intended for packaging applications, the examined samples were required to exhibit adequate mechanical strength and optimal surface properties. Therefore, bleached virgin kraft pulp derived from both softwood and hardwood species was selected as the fibrous raw material. To determine the botanical origin and fiber composition of the pulp, a microscopic analysis was performed, as illustrated in Figure 2.

The microscopic analysis revealed that the samples consisted of approximately 80% bleached sulfate softwood pulp derived from pine and 20% bleached sulfate hardwood pulp derived from beech, both exhibiting the characteristic blue-violet staining with Herzberg’s reagent. This staining indicates a low residual lignin content (up to approximately 9%) and a high degree of delignification. The high degree of delignification promotes the formation of strong hydrogen bonds between adjacent cellulose fibers, resulting in a well-developed fiber network with good interfiber bonding. Consequently, the examined paper samples were expected to exhibit mechanical properties suitable for their intended packaging application.

**Figure 3:**
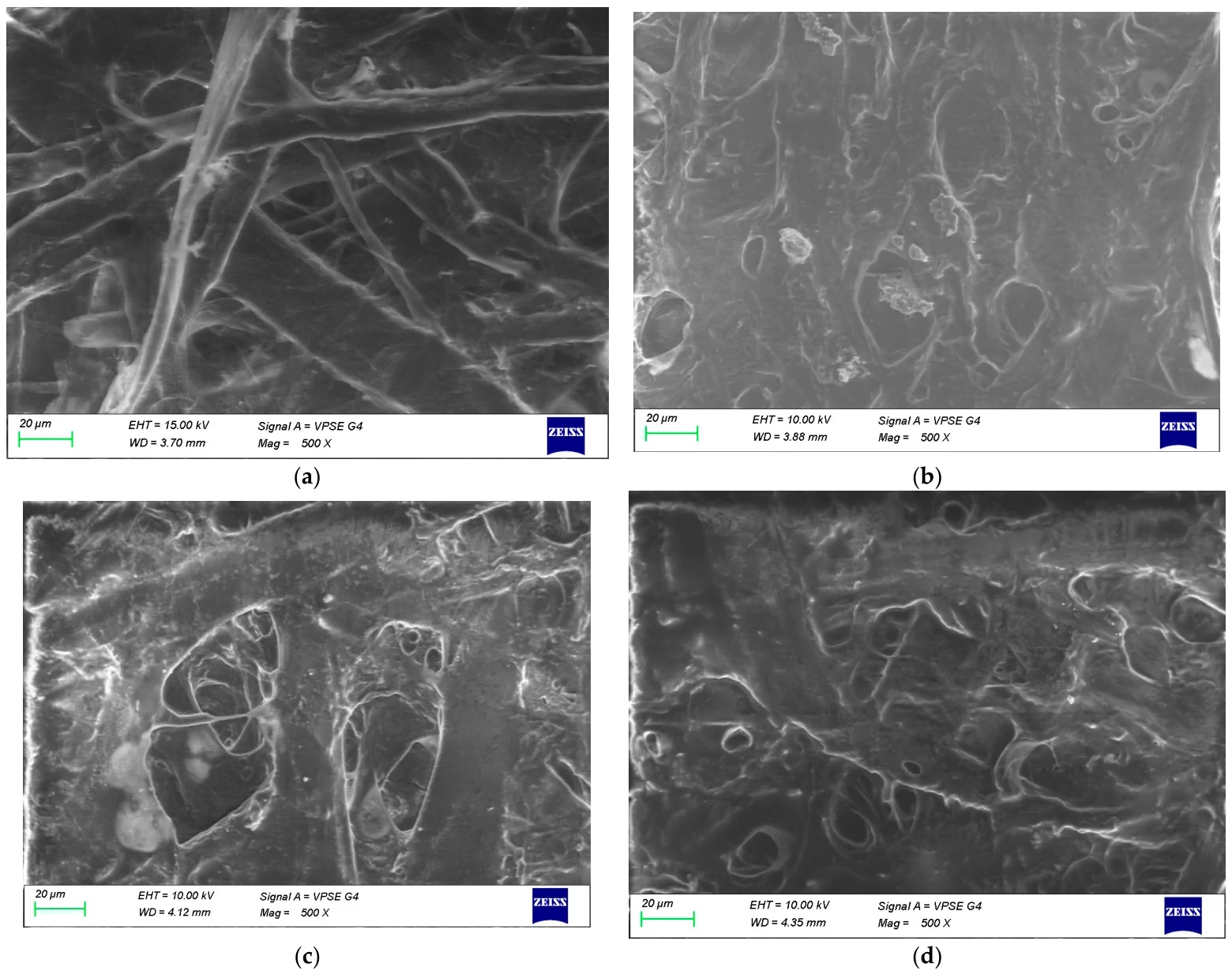
SEM micrographs taken at 500× magnification and operating at 10 kV voltage: (**a**) base paper (control); (**b**) propolis A; (**c**) propolis B; (**d**) propolis C.

SEM micrographs of the untreated (base paper) and the developed paper materials treated with the three extracts are presented in Figure 3. The observations confirmed the successful deposition of the propolis-derived extracts onto the paper surface, as the surface pores were uniformly covered by a thin coating layer. The treated samples exhibited a homogeneous surface morphology without visible cracks, pinholes, or coating agglomerates, indicating a uniform distribution of the extracts across the paper surface.

As an anisotropic material and on the basis of the used experimental methods determining the properties of the paper in different directions, the provenance of meaningful trends is a complex process, which starts with determining the basic weight, thickness, density, porosity and smoothness of the paper (Table 2). The smoothness of the papers is of essential importance for the surface-treated papers. It is also an indicator of a change in the structure of the paper surface.

The data presented in Table 2 demonstrate the stability of the structural properties of both the cellulose-only and base paper samples. The incorporation of wet-end chemical additives resulted in a slight decrease in paper density accompanied by a corresponding increase in porosity. At the same time, enhanced interfiber bonding promoted the formation of a more compact and uniform paper structure, which contributed to improved surface smoothness.

The mechanical properties presented in Table 3 confirm the expected improvement in all four evaluated parameters, namely the tensile index (Nm/g), tensile energy absorption (TEA) index (mJ/g), elongation at break (%), and tear index (mN·m^2^/g), for the base paper compared with the cellulose-only sample. This improvement can be attributed to the combined effect of the wet-end additives, namely alkyl ketene dimer (AKD), which enhanced the hydrophobicity of the paper, and modified polyacrylamide (PAA), which improved the retention of fibers and fines, thereby promoting the development of a stronger fiber network.

Further studies will focus on evaluating the physicochemical, mechanical, and other relevant properties of the paper following treatment with the ethanolic propolis extract.

### 3.2. Antimicrobial Activity of Paper Treated with Propolis Extracts

The amount of extract adsorbed onto the paper specimens (each with an area of 25 cm^2^) for the test microorganisms using the three propolis samples is presented in Table 4.

The extracts used to treat the paper penetrated its structure, and the average amount of adsorbed extract was: 1.03 mg/cm^2^ for sample No. 1; 1.04 mg/cm^2^ for sample No. 2; and 1.04 mg/cm^2^ for sample No. 3. These differences are attributed to the different origins of the propolis and the corresponding variations in its chemical composition. The average amount of extract retained on the paper is 1.04 mg/cm^2^. The data demonstrate that paper is a suitable matrix for the incorporation of propolis extract. As also reported by other authors, paper and other fibrous materials are suitable substrates for the incorporation of bioactive substances [57,58]. They are biodegradable, environmentally sustainable, and can be readily modified by coating or impregnation with active agents.

**Figure 4:**
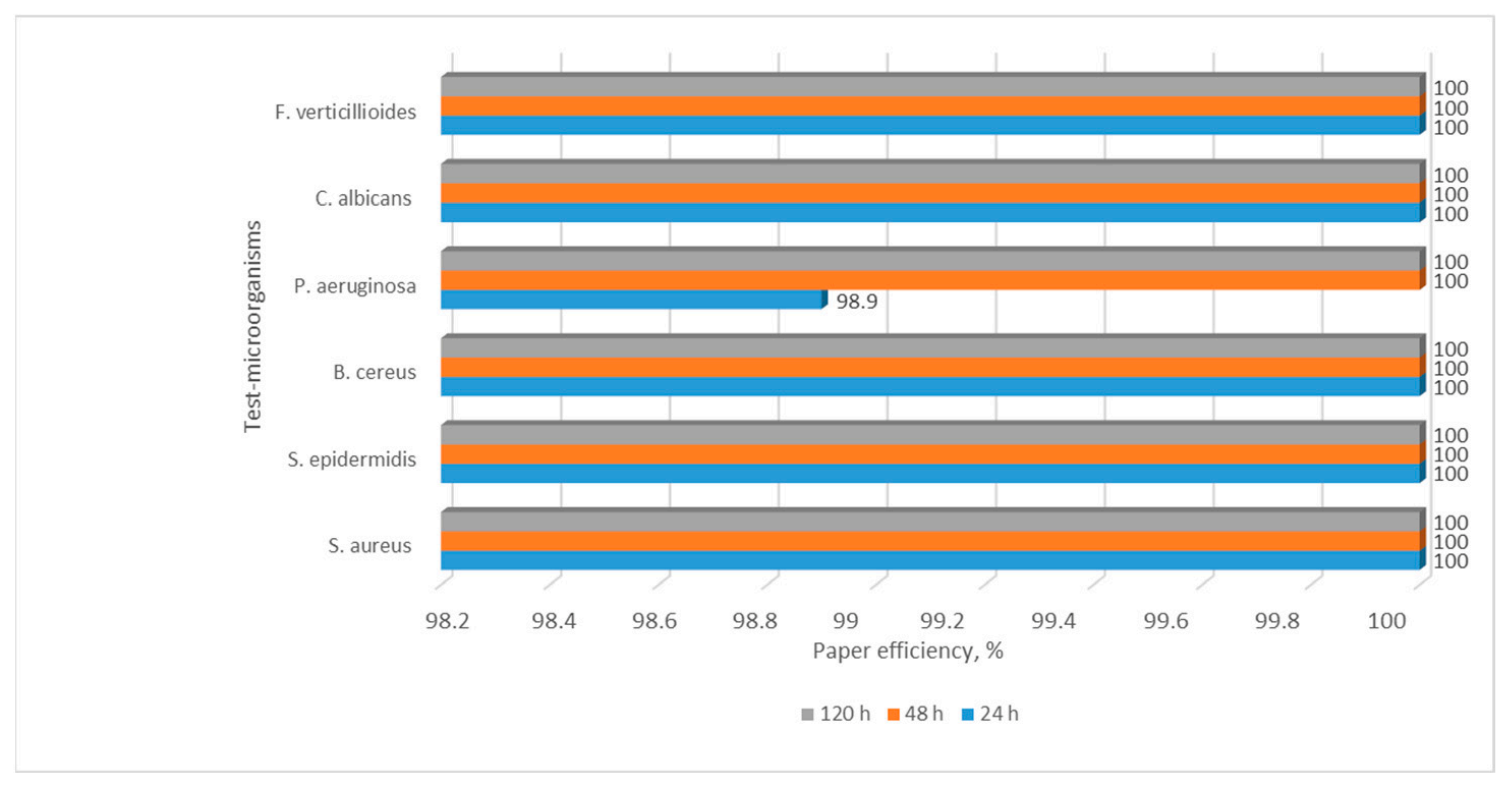
Antimicrobial activity of paper treated with extracts from propolis A stored for 30 years (change in value for bacteria *P. aeruginosa* 98.9 ± 0.01%).

**Figure 5:**
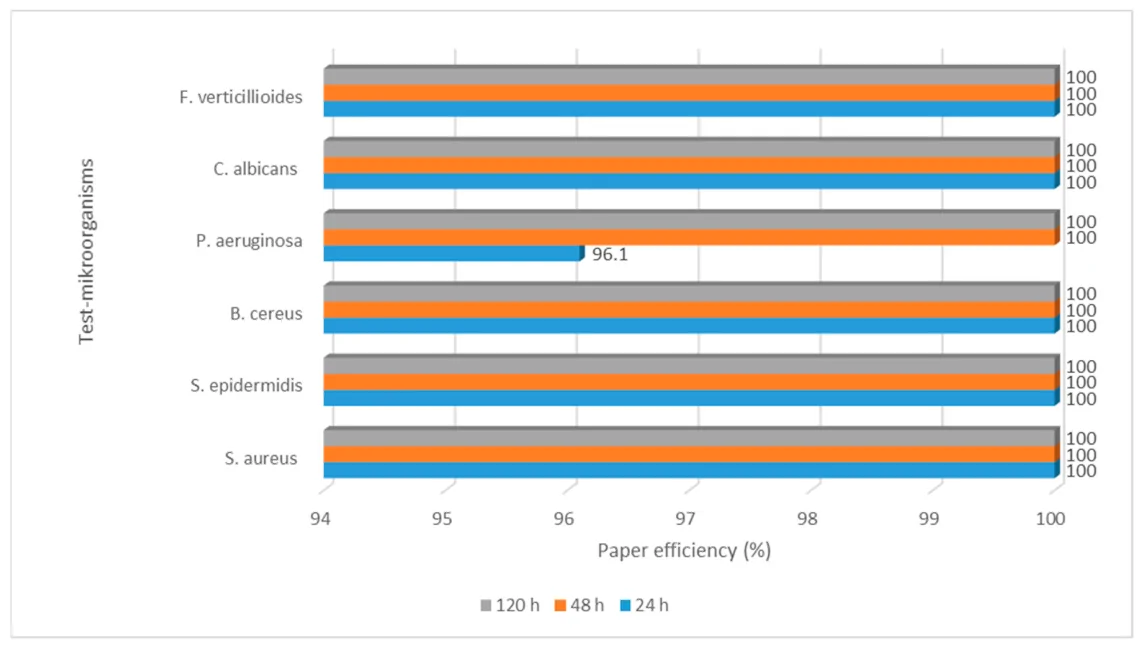
Antimicrobial activity of paper treated with extracts from propolis B from Central Bulgaria, harvest 2023 (change in value for bacteria *P. aeruginosa* 96.1 ± 0.01%).

**Figure 6:**
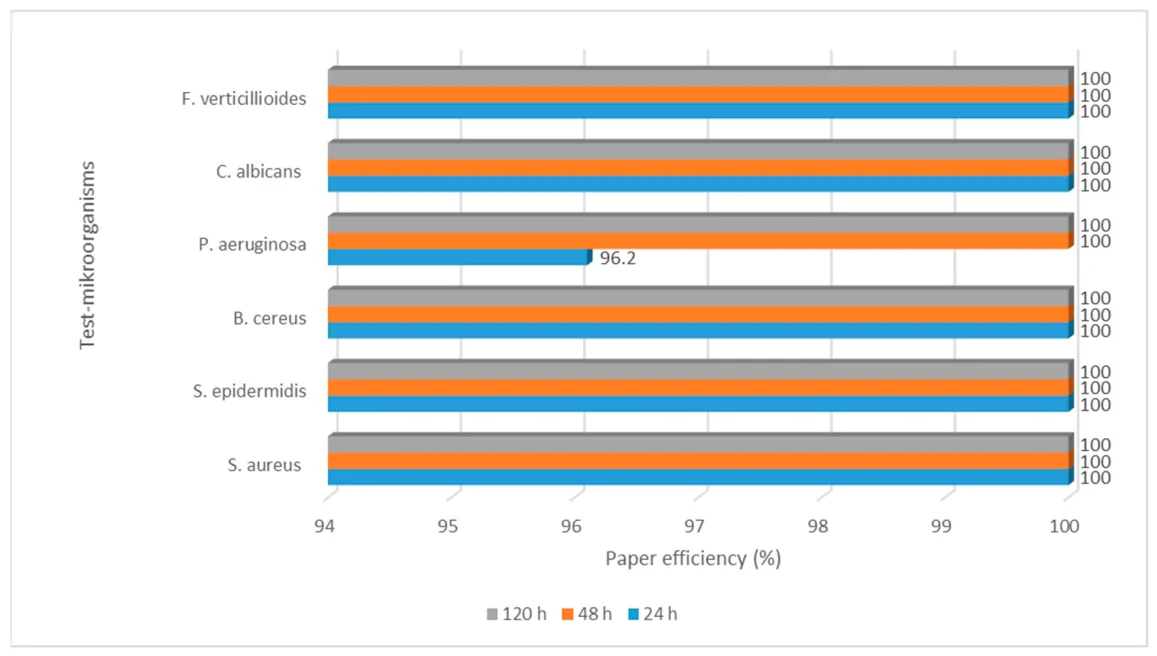
Antimicrobial activity of paper treated with extracts from propolis C from Northeastern Bulgaria, harvest 2023 (change in value for bacteria *P. aeruginosa* 96.2 ± 0.01%).

Figure 4, Figure 5 and Figure 6 present the antimicrobial activity of the treated paper with extracts from propolis stored for 30 years (propolis A), from Central Bulgaria (propolis B), and from Northeastern Bulgaria (propolis C), respectively.

The antimicrobial effect of the propolis-treated paper was detected immediately after sample inoculation (after 24 h), as well as after 48 and 120 h at room temperature (20 ± 0.5 °C). The active paper inhibited the growth of the Gram-negative bacteria *P. aeruginosa* by 98.9% (sample No. 1), 96.1% (sample No. 2), and 96.2% (sample No. 3). The overall antimicrobial efficiency of the paper against all tested microbial strains was 99.82%, 99.35%, and 99.37% for the respective samples (Figure 4, Figure 5 and Figure 6).

The difference in the efficiency of the paper samples treated with propolis extracts from Central and Northeastern Bulgaria is observed at 24 h of the treatment only against the Gram-negative bacteria *P. aeruginosa*.

The antimicrobial mechanisms of propolis have been extensively investigated and described by numerous authors. It damages the morphological structure of the cells by altering the permeability of the cell membrane and disrupting its integrity, resulting in cell lysis [59]. This leads to disturbances in the cellular energy metabolism by affecting the transport proteins responsible for the uptake of nutrients essential for cell survival. Propolis components that penetrate into the cytoplasm disrupt key metabolic pathways, inducing stress on intracellular homeostasis and inhibiting ATP synthesis [59]. Cell division is impaired through the inhibition of nucleic acid synthesis, thereby preventing the replication of microbial DNA and RNA [60,61].

Our results show that propolis, regardless of origin and storage time, is a suitable natural product for active food packaging.

These data confirm studies on the application of different natural products as antimicrobial active packaging materials [15,16,17,62,63,64].

The antimicrobial and antioxidant activities of propolis are attributed to flavonoids, phenolic acids and their esters, aromatic aldehydes, and ketones [65,66,67].

Flavonoids constitute the largest group of compounds in propolis. Their antioxidant activity is based on scavenging free radicals and interrupting oxidative chain reactions in lipids and proteins [20]. Their microbial inhibitory activity is exerted through the disruption of microbial membrane permeability and inhibition of cellular enzymes [68].

Phenolic acids (caffeic, sinapic, and ferulic acids), as well as their esters, exhibit strong antioxidant and antimicrobial activities. They act by chelating metal ions that catalyze oxidation and by directly interacting with bacterial membranes, thereby disrupting their integrity [69,70].

Aromatic aldehydes and ketones contribute to the biological activity of propolis through their antimicrobial effect and synergistic interactions with the principal bioactive groups in propolis, namely polyphenols and flavonoids [61,68].

It has been demonstrated that paper can serve as a carrier for propolis extracts while preserving the structural and functional properties of the packaging. Proper integration of propolis is essential to ensure the controlled release of bioactive substances, preserve the mechanical and barrier properties of the packaging, and minimize the impact of propolis on the taste and aroma of the food product.

## 4. Conclusions

Treating paper made from a cellulose mixture of bleached kraft woodfree pulp from softwood and hardwood species in an 80:20 ratio with propolis extracts is highly effective. The treated paper remained protected from the investigated microorganisms. While the year and location of propolis collection influence its chemical composition, the main components remain present, albeit in varying amounts. This consistency is crucial for the potential use of propolis extracts in developing active materials for packaging.

Propolis-modified paper has potential as a bioactive material for extending the shelf life and enhancing the safety of food products. The results demonstrated a treatment efficiency exceeding 90%, indicating that propolis has significant potential for incorporation into antimicrobial active materials for packaging. The development of active materials based on cellulose and propolis requires a comprehensive approach, including compliance with European legislation governing food contact materials, the specific requirements for functional packaging systems, the principles of good manufacturing practice, and the applicable industrial guidelines for paper-based materials.

Sensory evaluation of paper treated with an ethanolic propolis extract is an important aspect in assessing its potential application as a food packaging material. Such investigations will help determine the suitability of the developed paper material for specific types of food products, taking into account possible interactions between the packaging material and the sensory characteristics of the packaged food. These aspects will be addressed in future studies.

## Figures and Tables

**Table 1:** Distribution of the major groups of compounds in the investigated propolis samples [43,44,45].

Compounds, %	Sample 1	Sample 2	Sample 3
Alcohols	2.47	1.65	0.90
Organic acids	0.19	0.30	0.27
Benzoic acids	0.85	2.51	1.46
Hydrocinnamic acids and esters	17.15	25.69	31.07
Monosaccharides	2.95	13.59	28.72
Fatty acids	1.54	0.88	1.73
Flavanones and flavones	74.85	55.38	35.85

**Table 2:** Basic weight, thickness, density, porosity and smoothness of cellulose-only and base paper samples.

Paper Properties	Dimension	Only Cellulose	Base Paper
Basic weight	g/m^2^	50.79 ± 0.48 ^a^	50.58 ± 0.48 ^a^
Thickness	mm	0.079 ± 0.0 ^a^	0.081 ± 0.0 ^a^
Density	kg/m^3^	642.91 ± 6.20 ^a^	624.44 ± 6.20 ^a^
Porosity	%	57.14 ± 0.55 ^a^	58.37 ± 0.56 ^a^
Smoothness (Bekk, top side)	s	11.98 ± 0.10 ^a^	11.96 ± 0.10 ^a^

^a^ indices showing significant differences (*p* < 0.05) between the mean values in the rows.

**Table 3:** Strength properties of cellulose-only and base paper samples.

Sample	Tensile Index, Nm/g	TEA Index, mJ/g	Elongation, %	Tear Index, mN·m^2^/g
Only cellulose	63.9 ± 0.60 ^a^	1160 ± 10.48 ^a^	2.5 ± 0.02 ^a^	1.1014 ± 0.01 ^a^
Base paper	69.8 ± 0.65 ^b^	1452 ± 12.48 ^b^	3.0 ± 0.028 ^a^	1.1989 ± 0.01 ^a^

^a,b^ indices showing significant differences (*p* < 0.05) between the mean values in the column.

**Table 4:** Extract adsorbed onto paper, mg/cm^2^ for each test microorganism.

Test- Microorganism	Propolis A	Propolis B	Propolis C
*S. aureus*	1.00 ± 0.01 ^a^	1.04 ± 0.01 ^a^	1.02 ± 0.01 ^a^
*S. epidermidis*	1.04 ± 0.01 ^a^	1.04 ± 0.01 ^a^	1.06 ± 0.01 ^a^
*B. cereus*	1.02 ± 0.01 ^a^	1.05 ± 0.01 ^a^	1.04 ± 0.01 ^a^
*P. aeruginosa*	1.04 ± 0.01 ^a^	1.04 ± 0.01 ^a^	1.04 ± 0.01 ^a^
*C. albicans*	1.06 ± 0.01 ^a^	1.02 ± 0.01 ^a^	1.05 ± 0.01 ^a^
*F. verticillioides*	1.04 ± 0.01 ^a^	1.02 ± 0.0 ^a^	1.04 ± 0.01 ^a^

^a^ indices showing significant differences (*p* < 0.05) between the mean values in the column.

## Data Availability

The original contributions presented in this study are included in the article. Further inquiries can be directed to the corresponding author.

## References

[1] Brody A., Bugusu B., Han J., Sand C., McHugh T. (2008). Innovative food packaging solutions. J. Food Sci..

[2] Marsh K., Bugusu B. (2007). Food packaging—Roles, materials, and environmental issues. J. Food Sci..

[3] Han J. (2014). Innovations in Food Packaging.

[4] Realini C., Marcos B. (2014). Active and intelligent packaging systems for a modern society. Meat Sci..

[5] Appendini P., Hotchkiss J. (2002). Review of antimicrobial food packaging. Innov. Food Sci. Emerg. Technol..

[6] Versino F., Ortega F., Monroy Y., Rivero S., López O., García M. (2023). Sustainable and bio-based food packaging: A Review on past and current design innovations. Foods.

[7] Westlake J., Tran M., Jiang Y., Zhang X., Burrows A., Xie M. (2022). Biodegradable active packaging with controlled release: Principles, progress, and prospects. ACS Food Sci. Technol..

[8] Duda-Chodak A., Tarko T., Petka-Poniatowska K. (2023). Antimicrobial compounds in food packaging. Int. J. Mol. Sci..

[9] Vilela C., Kurek M., Hayouka Z., Röcker B., Yildirim S., Antunes M., Nilsen-Nygaard J., Pettersen M., Freire C. (2018). A concise guide to active agents for active food packaging. Trends Food Sci. Technol..

[10] Makwana S., Choudhary R., Kohli P. (2015). Advances in antimicrobial food packaging with nanotechnology and natural antimicrobials. Int. J. Food Sci. Nutr. Eng..

[11] Takala P., Vu K., Salmieri R., Lacroix M. (2013). Antibacterial effect of biodegradable active packaging on the growth of Escherichia coli, Salmonella typhimurium and Listeria monocytogenes in fresh broccoli stored at 4 degrees C. LWT-Food Sci. Technol..

[12] Triantafyllou V., Akrida-Demertzi K., Demertzi P. (2007). A Study on the migration of organic pollutants from recycled paperboard materials to solid food matrices. Food Chem..

[13] Rakchoy S., Suppakul P., Jiankarn T. (2009). Antimicrobial effects of vanillin coated solution for coating paperboard intended for packaging bakery products. Asian J. Food Agro-Ind..

[14] Wiastuti T., Khasanah L., Atmaka Kawiji W., Manuhara G., Utami R. (2016). Characterization of active paper packaging incorporated with ginger pulp oleoresin. 10th Joint Conference on Chemistry. IOP Conf. Ser. Mater. Sci. Eng..

[15] Kostova I., Lasheva V., Georgieva D., Damyanova S., Stoyanova A., Stefanov S., Gubenia O. (2020). Antimicrobial active packaging paper based on dill weed essential oil. Cellul. Chem. Technol..

[16] Kostova I., Lasheva V., Georgieva D., Damyanova S., Fidan H., Stoyanova A., Gubenia O. (2020). Characterization of active paper packaging materials with coriander essential oil (*Coriandrum sativum* L.). J. Chem. Technol. Metall..

[17] Kostova I., Lasheva V., Fidan H., Georgieva D., Damyanova S., Stoyanova A. (2020). Effect of clary sage (*Salvia sclarea* L.) essential oil on paper packaging materials. Ukr. Food J..

[18] Dimov V., Ivanovska N., Manolova N., Bankova V., Nikolov N., Popov S. (1992). Immunomodulatory action of propolis. IV. Prophylatic activity against Gram-negative infections and adjuvant effect of the water-soluble derivate. Vaccine.

[19] Damianova S., Dimitrov D. (1998). Aromatic products in Bulgarian propolis: II. Fixing ability. Riv. Ital. EPPOS.

[20] Kujumgiev A., Tsvetkova I., Serkedjieva Y., Bankova V., Christov R., Popov S. (1999). Antibacterial, antifungal and antiviral activity of propolis of different geographic origin. J. Ethnopharmacol..

[21] Murad J., Calvi S., Soares A., Bankova V., Sforcin J. (2002). Effects of propolis from Brazil and Bulgaria on fungicidal activity of macrophages against *Paracoccidioides brasiliensis*. J. Ethnopharmacol..

[22] Salomao K., Dantas A., Borba C., Campos L., Machado D., Aquino Neto F., de Castro S. (2024). Chemical composition and microbicidal activity of extracts from Brazilian and Bulgarian propolis. Lett. Appl. Microbiol..

[23] Boyanova L., Gergova G., Nikolov R., Derejian S., Lazarova E., Katsarov N., Mitov I., Krastev Z. (2005). Activity of Bulgarian propolis against 94 *Helicobacter pylori* strains in vitro by agar-well diffusion, agar dilution and disc diffusion methods. J. Med. Microbiol..

[24] Orsi R., Sforcin J., Funari S., Bankova V. (2005). Effects of Brazilian and Bulgarian propolis on bactericidal activity of macrophages against *Salmonella typhimurium*. Int. Immunopharmacol..

[25] Boyanova L., Kolarov R., Gergova G., Mitov I. (2006). In vitro activity of Bulgarian propolis against 94 clinical isolates of anaerobic bacteria. Anaerobe.

[26] Gardjeva P., Dimitrova S., Kostadinov I., Murdjeva M., Peychev L., Lukanov L., Stanimirova I., Alexandrov A. (2007). A study of chemical composition and antimicrobial activity of Bulgarian propolis. Folia Med..

[27] Machado G., Leon L., De Castro S. (2007). Activity of Brazilian and Bulgarian propolis against different species of *Leishmania*. Mem. Inst. Oswaldo Cruz.

[28] Slavov A., Trifonov A., Peychev L., Dimitrova S., Peycheva S., Gotcheva V., Angelov A. (2014). Biologically active compounds with antitumor activity in propolis extracts from different geographic regions. Biotechnol. Biotechnol. Equip..

[29] Tumbarski Y., Todorova M., Topuzova M., Gineva G., Yanakieva V., Ivanov I., Petkova N. (2023). Comparative study on physicochemical, antioxidant and antimicrobial properties of propolis collected from different regions of Bulgaria. J. Apic. Sci..

[30] Dantas A., Olivieri B., Gomes F., De Castro S. (2006). Treatment of Trypanosoma cruzi-infected mice with propolis promotes changes in the immune response. J. Ethnopharmacol..

[31] Draganova-Filipova M., Nikolova M., Mihova A., Peychev L., Sarafian V. (2014). A pilot study on the immunomodulatory effect of Bulgarian propolis. Biotechnol. Biotechnol. Equip..

[32] Vilhemova-Ilieva N., Nikolova I., Nikolova N., Petrova Z., Trepechova M., Holechek D., Todorova M., Topuzova M., Ivanov I., Tumbarski Y. (2023). Antiviral potential of specially selected Bulgarian propolis extracts: In vitro activity against structurally different viruses. Life.

[33] Bankova V., Popova M., Trusheva B. (2014). Propolis volatile compounds: Chemical diversity and biological activity: A Review. Chem. Cent. J..

[34] Bankova V., Popov S., Marekov N. (1983). A Study on flavonoids of propolis. J. Nat. Prod..

[35] Bankova V., Marekov N. (1984). Propolis: Composition and standardisation. Farmacija.

[36] Bankova V., Kuleva L. (1989). Phenolic compounds in propolis from different regions in Bulgaria. J. Anim. Sci..

[37] Damianova S., Dimitrov D. (1997). Aromatic Products in Bulgarian propolis: I. Technology, physical and chemical properties. Riv. Ital. EPPOS.

[38] Badria F., Fathy H., Fatehe A., Ahmed M., Ghazy M. (2018). Chemical and biological diversity of propolis samples from Bulgaria, Libya and Egypt. J. Apither..

[39] Popova M., Trusheva B., Bankova V. (2017). Content of biologically active compounds in Bulgarian propolis: A Basis for its standardization. Bulg. Chem. Commun..

[40] Segueni N., Boutaghane N., Asma S., Tas N., Acaroz U., Arslan-Acaroz D., Shah S., Abdellatieff H., Akkal S., Peñalver R. (2023). Review on propolis applications in food preservation and active packaging. Plants.

[41] Pu Y., Jiang H., Zhang Y., Cao J., Jiang W. (2023). Advances in propolis and propolis functionalized coatings and films for fruits and vegetables preservation. Food Chem..

[42] Rollini M., Mascheroni E., Capretti G., Coma V., Musatti A., Piergiovanni L. (2017). Propolis and chitosan as antimicrobial and polyphenols retainer for the development of paper based active packaging materials. Food Packag. Shelf Life.

[43] Nikolova I., Dincheva I., Taneva I., Pencheva M., Georgieva D., Damyanova S., Prodanova-Stefanova V., Kostova I., Stoyanova A. (2024). Chemical composition and antioxidant activity of propolis, stored for more than 30 years. BIO Web Conf..

[44] Damyanova S., Dincheva I., Kostova I., Taneva I., Prodanova-Stefanova V., Pencheva M., Nikolova I., Koleva Y., Stoyanova A. (2025). Propolis from Central Bulgaria: Chemical composition and biological activity. J. Environ. Prot. Ecol..

[45] Prodanova-Stefanova V., Kostova I., Pencheva M., Taneva I., Nikolova I., Damyanova S., Koleva Y., Dincheva I., Stoyanova A. (2024). Chemical composition and antioxidant activity of propolis from Razgrad. Oxid. Commun..

[46] Wang L., Weller C. (2006). Recent advances in extraction of nutraceuticals from plants. Trends Food Sci. Technol..

[47] (1979). Pulps—Laboratory Beating, Part 1: Valley Beater Method.

[48] (2001). Pulps—Determination of Drainability-Part 1: Schopper-Riegler methodTechnical Corrigendum 1.

[49] (2004). Pulps—Preparation of Laboratory Sheets for Physical Testing-Part 2: Rapid-Köthen Method.

[50] (2021). Paper, Board and Pulps—Fibre Furnish Analysis.

[51] (2019). Paper and Board—Determination of Grammage.

[52] (2022). Paper and Board—Determination of Thickness, Density and Specific Volume.

[53] (2002). Paper and Board—Determination of Smoothness (Bekk Method)-Technical Corrigendum 1.

[54] (2008). Paper and Broad—Determination of Tensile Properties. Part 2: Constant rate of elongation method (20 mm/min).

[55] (2012). Paper—Determination of Tearing Resistance—Elmendorf method.

[56] Zaika L. (1988). Spices and herbs: Their antimicrobial activity and its determination. J. Food Saf..

[57] Kóczán Z., Pásztory Z. (2024). Overview of natural fiber-based packaging materials. J. Nat. Fibers.

[58] Sarkhel S., Kaur S., Das R., Sharma A., Kheto A., Saha D., Kumar Y. (2026). Antimicrobial active packaging with biopolymers and natural extracts: Sustainable solutions and technological challenges. Sustain. Food Technol..

[59] Zhao Y., An J., Su H., Li B., Liang D., Huang C. (2022). Antimicrobial food packaging integrating polysaccharide-based substrates with green antimicrobial agents: A sustainable path. Food Res. Int..

[60] Bouchelaghem S. (2022). Propolis characterization and antimicrobial activities against *Staphylococcus aureus* and *Candida albicans:* A review. Saudi J. Biol. Sci..

[61] Pasupuleti V., Sammugam L., Ramesh N., Gan S. (2017). Honey, propolis, and royal jelly: A comprehensive review of their biological actions and health benefits. Oxid. Med. Cell. Longev..

[62] Rodríguez Y., Sánchez-Catalán F., Rojano B., Durango D., Gil J., Marín-Loaiza J. (2012). Caracterización fisicoquímica y evaluación de la actividad antioxidante de propóleos recolectados en el departamento del Atlántico, Colombia. Rev. UDCA Actual. Divulg. Científica.

[63] Kalkan H., Canbay E., Öztürk Ş., Aldemir O., Sözmen E. (2018). Biotransformation of propolis phenols by L. plantarum as a strategy for reduction of allergens. Food Sci. Biotechnol..

[64] Wyrwa J., Barska A. (2017). Innovations in the food packaging market: Active packaging. Eur. Food Res. Technol..

[65] Peixoto S., Nascimento A., Vicente C., Barros A. (2025). Solvent-driven extraction of bioactive compounds from propolis for application in food industry matrices. Appl. Sci..

[66] Osés S., Marcos P., Azofra P., de Pablo A., Fernández-Muíño M., Sancho M. (2020). Phenolic profile, antioxidant capacities and enzymatic inhibitory activities of propolis from different geographical areas: Needs for analytical harmonization. Antioxidants.

[67] Bankova V. (2005). Chemical diversity of propolis and the problem of standardization. J. Ethnopharmacol..

[68] Sforcin J., Bankova V. (2011). Propolis: Is there a potential for the development of new drugs?. J. Ethnopharmacol..

[69] Marcucci M. (1995). Propolis: Chemical composition, biological properties and therapeutic activity. Apidologie.

[70] Shao B., Mao L., Tang M., Yan Z., Shao J., Huang C., Sheng Z., Zhu B. (2021). Caffeic acid phenyl ester (CAPE) protects against iron-mediated cellular DNA damage through its strong iron-binding ability and high lipophilicity. Antioxidants.

